# Intravital FRET: Probing Cellular and Tissue Function *in Vivo*

**DOI:** 10.3390/ijms160511713

**Published:** 2015-05-21

**Authors:** Helena Radbruch, Daniel Bremer, Ronja Mothes, Robert Günther, Jan Leo Rinnenthal, Julian Pohlan, Carolin Ulbricht, Anja E. Hauser, Raluca Niesner

**Affiliations:** 1 Neuropathology, Charité–University of Medicine, Berlin 10117, Germany; E-Mails: Helena.radbruch@charite.de (H.R.); ronja.mothes@charite.de (R.M.); jan-leo.rinnenthal@charite.de (J.L.R.); Julian.pohlan@charite.de (J.P.); 2 Germany German Rheumatism Research Center, Berlin 10117, Germany; E-Mails: daniel.bremer@drfz.de (D.B.); r.guenther@drfz.de (R.G.); carolin.ulbricht@drfz.de (C.U.); hauser@drfz.de (A.E.H.); 3 Immundynamics and Intravital Microscopy, Charité–University of Medicine, Berlin 10117, Germany

**Keywords:** intravital FRET, multi-photon microscopy, fluorescence lifetime imaging, genetically encoded calcium indicators

## Abstract

The development of intravital Förster Resonance Energy Transfer (FRET) is required to probe cellular and tissue function in the natural context: the living organism. Only in this way can biomedicine truly comprehend pathogenesis and develop effective therapeutic strategies. Here we demonstrate and discuss the advantages and pitfalls of two strategies to quantify FRET *in vivo*—ratiometrically and time-resolved by fluorescence lifetime imaging—and show their concrete application in the context of neuroinflammation in adult mice.

## 1. Introduction

On the one hand, it is widely accepted that FRET is the most common phenomenon used to monitor cellular function on a molecular basis in optical imaging. This phenomenon has been extensively studied and used in cell cultures or extra-cellularly. As far as extracellular and *in vitro* conditions are concerned, we experienced the development of powerful, elaborate evaluation tools to accurately quantify FRET. Ratiometric techniques, including two or more detection channels [1,2], techniques based on acceptor photobleaching [3,4] or time-resolved techniques [5,6,7,8] (fluorescence lifetime imaging (FLIM) or time-resolved fluorescence anisotropy imaging (trFAIM)), have successfully been employed to probe molecular and cellular function *in vitro*.

On the other hand, understanding and treating diseases implies the understanding of the organism as a whole—a highly complex functional network of billions of cells interacting and communicating with each other during its lifetime. The specific function of each cell is modified and refined in a dynamic manner physiologically or pathologically. Most cellular processes are tightly regulated by a complex interplay of protein interactions, modifications, sub-cellular translocations and more. The temporal and spatial precision of these events is critical for the function of the whole. The development of probes for intravital FRET that offer spatio-temporal detection of these cellular events is necessary to examine these molecular mechanisms, in real time and in the genuine context: the living organism. In this way, cellular plasticity, crosstalk, metabolic functions and drug responses can be directly monitored. Various strategies based on FRET with two or more chromophores as partners for the resonance energy transfer have been proposed and applied [9,10,11,12]. In this context, the development of mouse models encoding FRET biosensors based on fluorescent proteins has opened new opportunities for the long-term monitoring of cell and tissue function in living organisms and disease models [13,14]. As an alternative, the lenti- or retroviral transfection of cells, followed by their transfer to the living animal is a faster solution [15], however, having the disadvantage of a relatively high cellular death and a short time span, in which the cells are visible *in vivo*. The main advantage of cell transfer or transplantation is given by the ability to thoroughly characterize the biosensors of interest first under *in vitro* conditions, *i.e.*, cell culture, before employing them *in vivo* [16].

Whereas multi-photon microscopy technology has been extensively used to probe cellular dynamics and interactions in living adult mice [17], probing cellular, tissue and organ function proved to be more difficult. Numerous technological advances are required to fully take advantage of FRET aiming at intravital quantification of cell function. Mainly ratiometric FRET techniques have been applied intravitally. In order to fulfill the requirements for dynamic intravital FRET quantification, calibration-free techniques are needed, such as FLIM [16,18].

Using the example of the *CerTN L15* mouse strain, we here comparatively investigate the power and the pitfalls of intravital ratiometric FRET, as well as of intravital FLIM-FRET. The *CerTN L15* mouse contains the *TN L15* construct, a troponin-C, FRET based calcium biosensor. The FRET construct *TN L15* contains Cerulean and Citrine as the donor and acceptor, respectively. Probing early neuronal dysfunction, before morphological changes occur, meaning early diagnosis, is demonstrated on the example of chronic neuroinflammation in these mice.

## 2. Results and Discussion

### 2.1. Ratiometric FRET: Intravital Calibration

The fluorescence intensity images of Cerulean and Citrine (donor and acceptor in the *TN L15* construct) were simultaneously acquired at an 850 nm excitation wavelength. The detection wavelength windows were set at 475 ± 20 nm for Cerulean and 535 ± 25 for Citrine. Under these conditions, the bleed through of Cerulean on the Citrine detection channel was a portion, α, of the detected Cerulean signal, whereas no bleed through of Citrine is expected in the Cerulean detection channel [19]. The direct excitation of Citrine at 850 nm is negligible [20]. Both α and the excitation cross-talk were additionally verified by simultaneously acquiring the fluorescence signal in a third channel 593 ± 20 nm, in healthy mice.

In order to exclude detector induced artefacts of the signal, we exchanged the (identical) detectors of the two channels. We measured on each channel, with both detectors, the same signal. Further, we applied the same gain on both channels, but corrected the Cerulean and Citrine signals, *I*_Cerulean_ and *I*_Citrine_, with the quantum efficiency η of the photomultiplier tube at 475 and 535 nm, respectively.

Thus, the FRET ratio is expressed in our case as:

(1) $$nF/donor=\left((I_{\text{Citrine}}-I_{\text{background}1})/{\text{η}}_{535}-\text{α}∙\left(I_{\text{Cerulean}}-I_{\text{background}2}\right)/{\text{η}}_{475}\right)/\left(I_{\text{Cerulean}}-I_{\text{background}2}\right)/{\text{η}}_{475}$$

or

(2) $$NFRET=\left((I_{\text{Citrine}}-I_{\text{background}1})/{\text{η}}_{535}-\text{α}∙\left(I_{\text{Cerulean}}-I_{\text{background}2}\right)/{\text{η}}_{475}\right)/\sqrt{(I_{\text{Citrine}}-I_{\text{background}1})∙\left(I_{\text{Cerulean}}-I_{\text{background}2}\right)/{\text{η}}_{535}∙{\text{η}}_{475}}$$

**Figure 1:**
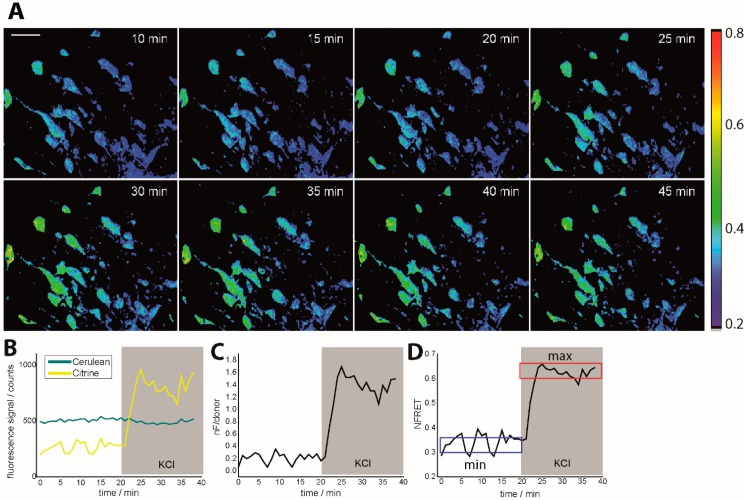
Intravital ratiometric FRET in healthy *CerTN L15* mice. (**A**) Time-lapse of NFRET ratio of a 300 × 300 × 70 µm^3^ region in the brain stem of a *CerTN L15* mouse before and during KCl treatment; (**B**) Cerulean and Citrine (donor and acceptor in *TN L15*) corrected averaged fluorescence signals during KCl treatment as well as averaged *nF*/donor ratio (**C**) and averaged NFRET ratio (**D**) time-evolutions. In (**D**), the blue frame defines the minimum NFRET ratio in healthy neurons, while the red frame defines the maximum NFRET ratio as given by treatment with KCl. The maximum NFRET ratio was validated by treatment with glutamate but is not the maximum reachable NFRET ratio as predicted by neurophysiology. The tolerance range for both minimum and maximum NFRET values is ≈0.1. *CerTN L15* mice are genetically encoding the FRET-based Ca^2+^-biosensor TN L15 in neurons. λ_exc_ = 850 nm, λ_em_ (Cerulean) = 475 ± 20 nm, λ_em_ (Citrine) = 535 ± 25 nm. Scale bar = 50 µm. All images are represented as *xyz* projections.

The values of *I*_background1_ and *I*_background2_ were calculated as mean values of signal within blood vessels (as well as defined areas without Thy1 expression), for each image. We employed two normalization strategies: (i) with respect to the donor signal (*nF*/donor, Equation 1); and (ii) to both signals of the donor and acceptor (NFRET) [21,22,23]. This resulted in different dynamic ranges of the normalized ratio, as expected, but all led to the same relative increase between healthy neurons and neurons treated with KCl solution (Figure 1, Movie S1). In order to calculate Δ*R*/*R* in %—usually given in intravital FRET studies [14,15,24]—we refer to the mean values of healthy neurons measured under intravital conditions as the minimum ratio and to the values achieved under calcium saturation in neurons as the maximum ratio. To determine the ratio value at saturation of neuronal calcium, we locally applied on the tissue in the operation field KCl solution (300 µM solution) or glutamate solution (100 µM solution). It is known from electrophysiological studies that both glutamate and KCl do not lead to complete saturation (100% calcium influx) and that 70% of calcium influx can be expected. Ionomycin is more adequate to reach true saturation (100%); however, in the necessary high concentrations it induces fast cell death and is not suitable to be used under intravital conditions.

Ratiometric FRET measurement under intravital conditions is a robust method to acquire relative changes of the parameter of interest, in our case, Calcium concentration. Thus, it is principally not possible to probe the true, absolute calcium influx in neurons under any conditions. The technique relates the pathological data to qualitative, thus uncertain references like healthy state and reaction to chemicals (KCl, glutamate, *etc.*).

We used the herein described FRET evaluation setup to measure pathologic neuronal calcium increase in chronic neuroinflammation, as well as the effect of various calcium inhibitors (MK801, phenytoin, NBQX or nifedipine), as discussed in previous studies [24].

### 2.2. Challenges in Intravital Ratiometric FRET

We verified the possibility of detecting the Cerulean and Citrine signals on four (narrower) channels, *i.e.*, spectral unmixing, as proposed by Ducros *et al.* [25] under the conditions required by intravital microscopy; that means FRET measurements in 3D over time with a time resolution of 30–60 s, per 300 × 300 × 50 µm^3^ (512 × 512 × 26 voxel). The resulting signal-to-noise ratio was insufficient to calculate the FRET ratio at an acceptable photon flux, *i.e.*, not a sufficient signal in each detection channel, at which the laser itself still does not induce a pathologic calcium increase in neurons, massive photobleaching or other tissue photodamage. The use of additional detection channels in the range 40–50 nm (three/four instead of two) did not lead to any additional accuracy increase in calculating the ratios (*nF*/donor or NFRET) and, additionally, would interfere with the detection of other relevant cellular compartments, like different immune cell subsets.

The limitations in terms of laser power and acquisition speed imposed by the intravital microscopy makes the use of the various highly elaborated FRET techniques (developed for cell culture measurements) inappropriate.

Despite a successful use of ratiometric FRET under specific conditions, several factors limit its applicability to deep-tissue *in vivo* functional imaging. The pitfalls of intravital ratiometric FRET are related in the first line to effects of a variable signal-to-noise ratio (SNR) over the 3D images of the donor and acceptor signals, respectively. Additionally, photobleaching may limit the application range of intravital ratiometric FRET. Here, we investigate these effects and identify the experimentally appropriate conditions to minimize them.

**Figure 2:**
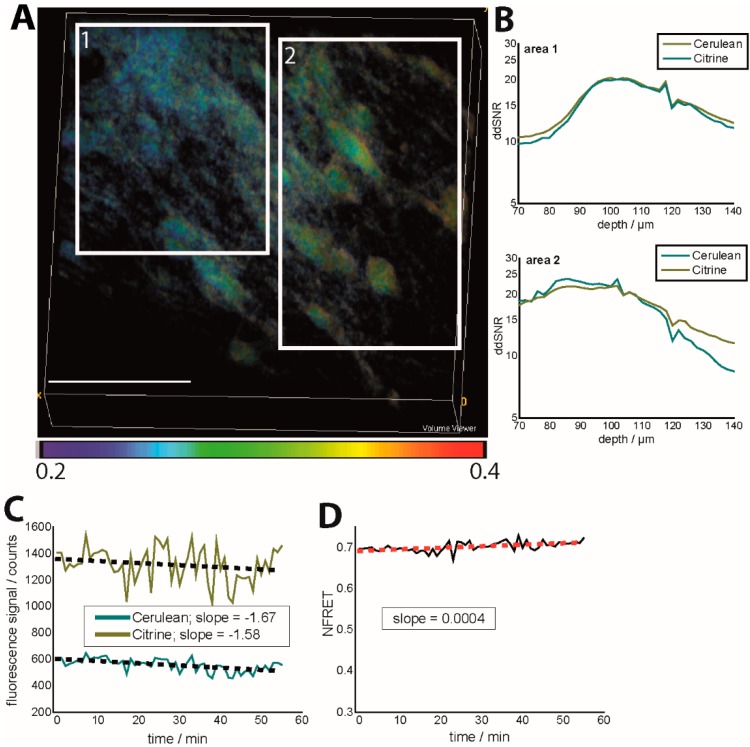
Effects of depth-dependent SNR and photobleaching on the NFRET ratio in the brain stem of *CerTN L15* mice (intravital imaging in healthy and untreated tissue). (**A**) NFRET ratio 3D image (300 × 300 × 70 µm³). Regions of different depth-dependent signal-to-noise ratios (ddSNR) show different NFRET ratios although the expected neuronal calcium is expected to be the same; (**B**) Dependence of ddSNR on imaging depth for both Cerulean and Citrine fluorescence signals in the two areas labeled in (**A**); (**C**) Time-dependence of the fluorescence signals of Cerulean and Citrine in Movies S2 and S3 and linear approximation of the photobleaching-ind uced time-decay; (**D**) Time-dependence of the corresponding NFRET ratio (Movie S4) as a consequence of photobleaching. λ_exc_ = 850 nm, λ_em_ (Cerulean) = 475 ± 20 nm, λ_em_ (Citrine) = 535 ± 25 nm. The mean laser power was 8 mW at 850 nm and a 160-fs pulse width, as well as an 80-MHz laser repetition rate.

As depicted in Figure 2A, different regions of a 3D image of 300 × 300 × 70 µm^3^ (517 × 517 × 36 voxel) are characterized by different signal-to-noise ratios (SNR), both in the Cerulean channel and in the Citrine channel. Moreover, there are severe differences between the depth-dependent SNR (ddSNR) of Citrine as compared to Cerulean at various sites due to the differential scattering behavior of the two fluorescent proteins. The experiments were performed in a healthy mouse, in which we expect the average neuronal calcium concentration to amount to approx. 100 nM all over the tissue. The variable ddSNRs of Cerulean and Citrine lead to a variation of the FRET ratio all over the image (Figure 2A). The solution to avoid such effects is to choose tissue areas of a homogeneously distributed fluorescence signal for both Cerulean and Citrine and to perform the KCl or glutamate calibration in each subject (mouse), which is not advisable if you want to investigate your probe without biased from artificial excitatory events. The depth-dependent SNR is calculated for each imaging depth as the ratio between the fluorescence signal within cellular structures (the background being subtracted) and the full-width at half maximum of the background distribution, *i.e.*, background noise. The background is considered to be the detected signal within blood vessels and is calculated for each image.

We demonstrate that different photobleaching of Cerulean and Citrine during 1 h of acquisition, every minute, had a negligible effect on the FRET ratio, if the mean laser power was kept under 3 mW under the objective lens (peak photon flux 10^28^ photons/cm^2^ s at 850 nm). However, considering a simple (gross) linear approximation for photobleaching, we could still retrieve a higher photobleaching rate for Cerulean as compared to Citrine. The slope of the fitting curve was −1.58 ± 0.54 for Citrine and −1.67 ± 0.34 for Cerulean, while the NFRET value increased over time with a slope of 4 × 10^−4^ (Figure 2B,C). Since it is known that photobleaching dependence on laser power is highly non-linear, we expect this effect to strongly affect the FRET signal at only slightly higher laser powers or over longer acquisition time windows.

### 2.3. Intravital FRET by Donor FLIM: Calibration-Free FRET Quantification

As discussed by Paul French’s lab [26], time-resolved techniques, such as FLIM, do not need any precedent calibration regarding the experimental conditions (laser power, detection efficiency, *etc.*) to determine the boundary conditions for the FRET ratio, which are usually related to a high uncertainty and variability. So far, FRET-FLIM is a calibration-free technique of particular relevance for intravital microscopy. By acquiring only the donor fluorescence signal (in our case, Cerulean), the effects of differently varying SNRs of Cerulean and Citrine are additionally avoided. However, measuring the dependence of FRET ratio by FRET-FLIM on the vital parameter of interest, in our case free calcium concentration, is required (Figure 4A).

In intravital or deep-tissue FRET measurements by donor FLIM, retrieving the cause of multiexponential decays of the donor leading to artefacts in the FRET ratio, such as photo-isomerization, represents a new challenge [27]. The acquisition of both donor and acceptor fluorescence decays leads back to unpredictable effects of a variable ddSNR also affecting the evaluation of the decay curves. New FRET constructs based on fluorescent proteins (as proposed by several groups in the last few years) with a well-characterized, mono-exponential fluorescence lifetime of the donors are one possible solution for this challenge [28,29]. Further efforts are currently being done (and are necessary) to translate the expertise on these constructs—gained under *in vitro* conditions—to transgenic mouse technology.

**Figure 3:**
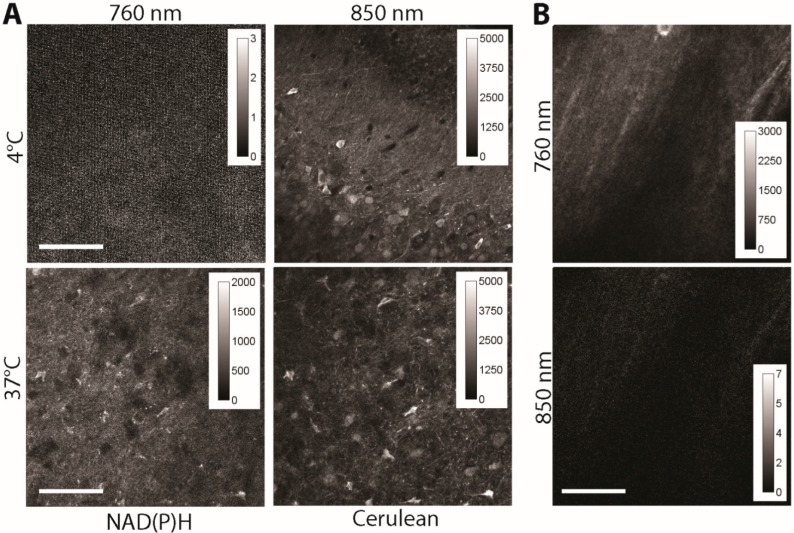
(**A**) Fluorescence signal of 300 × 300 µm areas in acute (live) brain slices of *CerTN L15* mice as measured at 760 and 850 nm, respectively. Detection at λ_em_ = 460 ± 30 nm. The temperature was set at 4 and 37 °C, to decrease the NAD(P)H metabolism at a minimum (4 °C) and to increase it at physiological levels (37 °C), respectively. Since at 4 °C only free NAD(P)H of a much shorter fluorescence lifetime is detected, the fluorescence intensity under excitation at 760 nm is very low (NAD(P)H fluorescence representing the integral under the decay curve), whereas under excitation at 850 nm it appears at high levels (Cerulean). At 37 °C NAD(P)H is mostly involved in metabolic processes, *i.e.*, is bound to enzymes, and shows an up to 10 times longer fluorescence lifetime as free NAD(P)H. Under these conditions, both under excitation at 760 nm and at 850 nm, high fluorescence signals are detected. The laser peak photon flux at the sample was kept constant for all experiments to ensure an accurate comparison; (**B**) Fluorescence signal of 300 × 300 µm^2^ areas in acute (live) brain slices of *C57/B6* mice (wild-type) as measured at 760 and 850 nm, respectively. Detection at λ_em_ = 460 ± 30 nm. Scale bar = 100 µm.

Here, we investigate the contribution of an additional effect that may cause severe artefacts on FRET quantification: the autofluorescence of NADH and NADPH to the fluorescence signal of Cerulean, which would lead under intravital conditions to an artificially lower FRET signal since enzyme-bound NAD(P)H has a fluorescence lifetime of approx. 2 ns. We investigated acute brain slices of adult CerTN L15 mice kept at 4 °C (low metabolic activity, mostly free NAD(P)H, fluorescence lifetime ≈ 400 ps) and at 37 °C (mostly enzyme-bound NAD(P)H, fluorescence lifetime ≈ 2000 ps). The samples were subsequently excited at 760 nm (for NAD(P)H) and at 850 nm (for Cerulean). The fluorescence signal was detected at 460 ± 30 nm. Since at 4 °C, the fluorescence signal could be detected only after excitation at 850 nm, we conclude that there is no or negligible cross-talk between Cerulean and NAD(P)H detection and that FRET quantification is not affected by auto-fluorescence (Figure 3A). The fact that in the brain tissue of C57/B6 mice (wild-type mice), any specific fluorescence signal is detected after the excitation at 850 nm, in the channel, 460 ± 30 nm is supportive for this conclusion (Figure 3B). A possible influence of flavoproteins is excluded, since they are emitting at longer wavelengths (green part of the spectrum).

**Figure 4:**
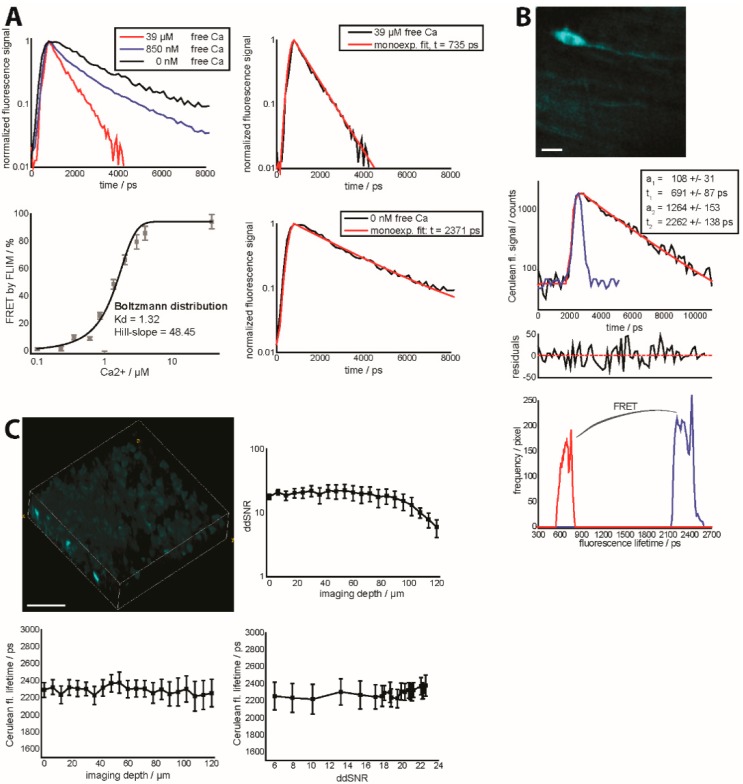
(**A**) Fluorescence decay curves (**right**, **top**) of the isolated construct TN L15 at 0 nM, 850 nM and 39 µM free calcium. The extreme conditions, *i.e.*, 0 nM and 39 µM free calcium are additionally mono-exponentially approximated (**left**, **top** and **bottom**). Calibration curve of the biexponential FLIM-FRET ratio of the isolated construct TN L15 at various free calcium concentrations (**right**, **bottom**). *a*_1_ and *a*_2_ are the relative concentrations of FRET-quenched and unquenched Cerulean as given by the biexponential model of the fluorescence decay curve (Supplementary Material); (**B**) Fluorescence signal of Cerulean of a 150 × 150 × 20 µm^3^ (256 × 256 × 11 voxel) in the brain stem of a healthy *CerTN L15* mouse (intravital measurement). The graph in the middle depicts an example of a decay curve in one of the brightest pixels of the image, including the biexponential approximation curve reconvolved with the instrument response function (blue curve), together with the final parameters and the corresponding residuals. The lowest graph shows the distributions of the fluorescence lifetimes of unquenched and FRET-quenched Cerulean over the 3D image at the top. Scale bar = 25 µm; (**C**) 3D fluorescence intensity image of Cerulean (**right**, **top**) in a healthy (live) brain slice of a CerTN L15 mouse (300 × 300 × 120 µm^3^, 517 × 517 × 40 voxel). ddSNR curve (**left**, **top**) as well as corresponding dependence of Cerulean fluorescence lifetime on imaging depth (**right**, **bottom**) and ddSNR (**left**, **bottom**). Detection at λ_em_ = 460 ± 30 nm. Excitation at λ_exc_ = 850 nm, mean laser power 8 mW.

One of the main challenges in intravital FLIM is finding the appropriate experimental conditions to fulfill the requirements of biological and biomedical applications. These are repeated acquisition of the same organ field of view for imaging cellular dynamics, typically 150 × 150 × 20 to 500 × 500 × 100 µm^3^ (256 × 256 × 11 to 1024 × 1024 × 51 voxel), every 15–60 s, low/negligible photodamage, achievable only at low mean and peak excitation powers on the order of 1–10 mW (peak photon flux 10^28^ photons/cm^2^ s, slightly varying with excitation wavelength and pulse width) and multi-color acquisition, which allow for investigating the orchestrated interplay of different cellular compartments [7,30]. These requirements are in conflict with the acquisition of a high fluorescence signal, desirable for an artefact-free exponential evaluation of fluorescence decays. By using our parallelized TCSPC device (electronics dead-time 5.5 ns and parallelized photoelectron gain [18]), we could repeatedly acquire FLIM images that we could biexponentially evaluate in a robust, reliable manner. Figure 4B depicts a typical intravital 3D FLIM stack (256 × 256 × 11 voxel, 150 × 150 × 20 µm^3^) acquired within 15 s, an example of a biexponentially-evaluated pixel decay curve after 2 × 2 pixel Gaussian noise filtering and the distribution of the short (FRET-quenched) and long (unquenched, genuine Cerulean) fluorescence lifetimes over the whole 3D stack. The mean power was 8 mW (5 × 10^28^ photons/cm^2^ s at 850 nm) in our case. Figure 4A depicts the dependency of the FRET ratio measured by FLIM on the calcium concentration, under extracellular conditions, *i.e.*, the isolated TN L15 construct, using the same setup as employed for intravital experiments. Under intracellular, *in vitro* as well as *in vivo* conditions, we could not observe the high FRET ratios acquired at a high free calcium concentration. This is due to the fact that free calcium concentrations even in the lower µM range are not possible in the case of live imaging. Additionally, Figure 4A depicts fluorescence decay curves at various free calcium concentrations together with the mono-exponential approximations of pure unquenched Cerulean (0 nM free calcium) and FRET-quenched Cerulean (39 µM free calcium). The fluorescence lifetime of unquenched Cerulean agrees with that of CFP ubiquitously expressed under the β-actin promoter in mice as measured by us [18] and with the results of others [31].

We previously showed that differential scattering behavior of Cerulean and Citrine, respectively, leads to differential ddSNRs for the two fluorescent proteins and, thus, to an unreliable FRET ratio in deep-tissue and intravital imaging. In the case of deep-tissue and intravital FRET-FLIM, we could demonstrate that the fluorescence lifetime of EGFP expressed in neurons (Thy1 mouse) can be reliably retrieved at various imaging depths, in brain tissue, as long as the ddSNR is higher than 4 [18]. Here, we performed similar experiments on brain slices of healthy CerTN L15 mice and show (Figure 4C) that the fluorescence lifetime of Cerulean can be correctly measured at various imaging depths and at various ddSNR above a value of 5.

### 2.4. Intravital FLIM-FRET in Experimental Autoimmune Encephalomyelitis

During chronic neuroinflammation, we expect a severe increase of neuronal calcium to precede long-term, reversible or irreversible morphological changes of the neuronal processes and somata.

In order to demonstrate the power of intravital FRET-FLIM, we induced experimental autoimmune encephalomyelitis (EAE) in *CerTN L15* mice with LysM^+^ tdRFP expressing cells (immune cells of myeloid origin). At the peak of disease, the immune cells invade the central nervous system and build the specific lesions, similar to the human disease multiple sclerosis, at which we expect neuronal dysfunction and, finally, neuronal death at the lesion site.

**Figure 5:**
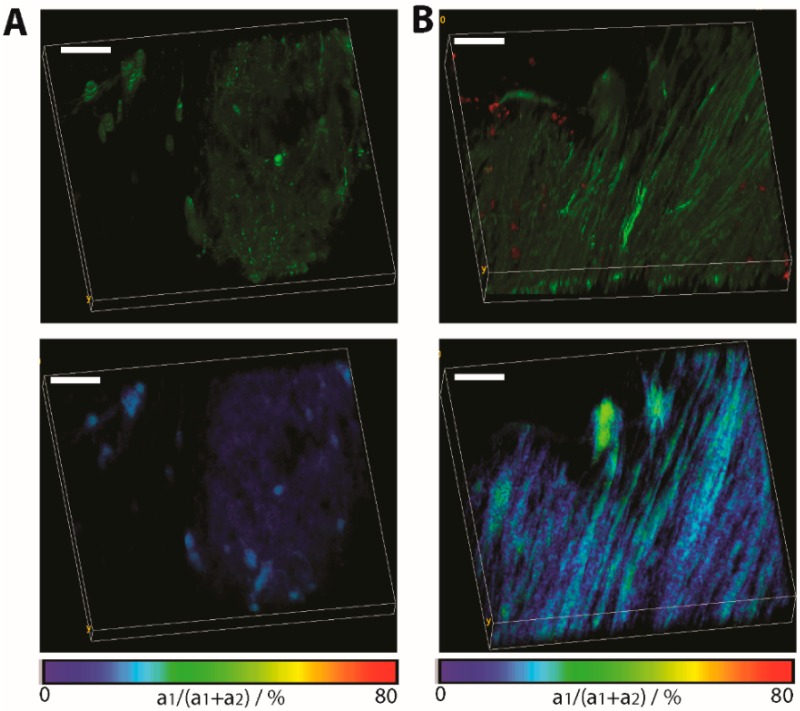
Application of FRET-FLIM in chronic neuro-inflammation to probe neuronal dysfunction. (**A**) Fluorescence intensity image (**top**) and FLIM-FRET ratio image (**bottom**) in a normal appearing region in the brain stem of a *CerTN L15 x LysM tdRFP* mouse. In the top image, certain subsets of neurons appear green; (**B**) The same images (intensity and FLIM-FRET ratio image) in a region of the brain stem invaded by immune cells (red). Image dimensions: 300 × 300 × 40 µm^3^ (517 × 517 × 21 voxel). λ_exc_ = 850 nm. λ_em_ = 460 ± 30 nm. Scale bar = 50 µm. As expected, at regions not affected by inflammation, the FRET ratio indicates physiological levels of neuronal calcium, while at the lesion site, the neuronal calcium increases at sustained pathological values.

Figure 5 depicts the differences between normal appearing tissue and lesion sites in the brain stem of *CerTN L15 x LysM^+^ tdRFP* mice affected by EAE, as shown by the accumulation of immune cells (red). In this case, it is evident that the increased neuronal calcium over the limit of 1 µM appears only at the inflammation sites. Electrophysiological measurements in primary neuronal cultures identified a sustained 1 µM intracellular calcium concentration to lead to cell death [32].

## 3. Experimental Procedures

### 3.1. In Vivo Two-Photon Laser-Scanning Microscopy (TPLSM)

The *in vivo* FRET experiments were performed using a specialized two-photon laser-scanning microscope based on a commercial scan head (TriMScope, LaVision BioTec, Bielefeld, Germany). We used for detecting the fluorescence signal photomultiplier tubes in the ranges 460 ± 30 or 475 ± 25 (blue range), 535 ± 25 (green range) and 593 ± 20 nm (red range). Alternatively, we used to detect fluorescence in a time-resolved manner a 16-channel parallelized TCSPC detector (FLIM-X_16_, LaVision BioTec, Bielefeld, Germany) in the range 460 ± 30 nm. The excitation wavelength of Cerulean was 850 nm (detection at 460 ± 30 or at 475 ± 25 nm), of NADH and NADPH 760 nm (detection at 460 ± 30 nm) and of tdRFP in immune cells 1110 nm (detection at 593 ± 20 nm). Citrine was detected at 535 ± 25 nm.

For both intensity and fluorescence lifetime imaging we used an average maximum laser power of maximum 8 mW to avoid photodamage. The experimental parameters for FLIM were 160-ps histogram bin and a maximum acquisition time for a 512 × 512 image of 5 s to record a fluorescence decay stack. The time-window in which the fluorescence decays were acquired was set to 9 ns.

### 3.2. Data Analysis

FLIM data analysis was performed using self-written software based on Levenberg–Marquardt algorithms for non-linear and bilinear fitting (RINIFLIM) [18]. In order to account for the effect of the instrument response function, we performed re-convolution after each iteration step during the bi-exponential approximation of the Cerulean fluorescence decay. The instrument response function was measured as the time-resolved second-harmonics generation signal of KH_2_PO_4_ powder. Statistical analysis and graphical presentation were carried out with OriginPro (OriginLab, Northampton, MA, USA). 3D image reconstruction was performed using Fiji/ImageJ or Volocity (Perkin Elmer, Rodgau, Germany).

### 3.3. Mice

The *CerTN L15 x LysM tdRFP* mouse expresses a FRET-based calcium biosensor in subsets of neurons (Thy1 expression cassette) consisting of Cerulean (donor) and Citrine (acceptor) bound to troponin C, a calcium-sensitive protein [14]. Additionally, tdRFP is expressed in LysM+ cells using Cre-loxP technology [33,34]. The mice are bred on a C57/Bl6 background.

### 3.4. Experimental Autoimmune Encephalomyelitis (EAE)

EAE was induced as previously described [24,35]. Briefly, mice were immunized subcutaneously with 150 µg of MOG_35–55_ (Pepceuticals, Leicestershire, UK) emulsified in CFA (BD Difco, Heidelberg, Germany) and received 200 ng pertussis toxin (PTx, List Biological Laboratories, Inc., Campbell, CA, USA) intraperitoneally at the time of immunization and 48 h later.

### 3.5. Preparation of the Brain Stem Window for Intravital Imaging

The preparation of the imaging field was similar to our previous description. Animal experiments were approved by the appropriate state committees for animal welfare (G0198/11 and G0181/10, LAGeSo—Landesamt für Gesundheit und Soziales) and were performed in accordance with current guidelines and regulations.

## 4. Conclusions

The possibility of quantifying FRET in living organisms opens unprecedented insight into the dynamic nature of cellular function in its genuine context and, thus, is invaluable for biomedical research. While ratiometric intravital FRET offers the possibility of increased acquisition speed, it allows only for a relative measure of function in tissue, since it necessitates previous *in situ* calibration due to differential scattering (and ddSNR) of the donor and acceptor, respectively. This is a highly difficult requirement for intravital imaging and not feasible for disease models. FLIM allows for calibration-free measurements, *i.e.*, scattering and, thus, ddSNR independent measurements, and is the method of choice for highly accurate organ function quantification under pathogenic conditions with the absence of non-excited values. However, since higher signals are required in FLIM, the risk of photodamage is higher or the acquisition speed is lowered as compared to ratiometric FRET. If these considerations are taken into account the choice of one or the other technique to perform FRET intravitally is determined by the requirements of the specific biomedical application.

## Supplementary Materials

Supplementary materials are available at: www.mdpi.com/1422-0067/16/05/11713/s1.
