# Supplementary material for: Intravital FRET: Probing Cellular and Tissue Function in Vivo
Source: Int J Mol Sci. 2015 May 21;16(5):11713–27. doi: 10.3390/ijms160511713 (PMC4463726; doi:10.3390/ijms160511713)
Supplement: Supplementary file 1 [file ijms-16-11713-s001.zip › ijms-79058-Supplementary Information/ijms-79058-Supplementary Information.pdf]

Supplementary Information

Titration of TN L15 construct by FRET FLIM

The TN L15 construct is a FRET-based construct containing the calcium sensitive molecule Troponin C. Troponin C has two high affinity and two low affinity calcium binding sites, which are also sensitive to magnesium but at higher concentrations (mM range). The FRET pair, *i.e.*, Cerulean as donor and Citrine as acceptor, is bound to Troponin C in such a way, that calcium binding at all four sites is detectable by FRET. Hence, the titration curve of the FRET ratio for the TN L15 construct is governed by a double Boltzmann distribution, characterized by two K_d values—one for the high affinity and one for the low affinity binding sites. Both by ratiometric FRET and by FRET FLIM, only one effective K_d (only a single averaged Boltzmann distribution) can be acquired.

The equation we used for the approximation of the titration of TN L15 by FRET FLIM in Figure 4A is:

$$FRET = FRET_{\max} + (FRET_{\max} - FRET_{\min})/(1 - e^{\mathrm{Hill}\,([Ca]-Kd)}) \quad (1)$$

Biexponential approximation in FRET FLIM

The fluorescence decay curve of the Cerulean as FRET donor of TN L15 can be modeled by a biexponential decay function:

$$F(t) = a_1 \cdot e^{-(t-t0)/\tau 1} + a_2 \cdot e^{-(t-t0)/\tau 2} \quad (2)$$

with $F(t)$ the time-resolved fluorescence signal, τ_1 the fluorescence lifetime of Cerulean entirely quenched by FRET, τ_2 the fluorescence lifetime of unquenched Cerulean and a_1 and a_2 the concentration of FRET-quenched and unquenched Cerulean, respectively. Hence, the ratio $a_1/(a_1 + a_2)$ represents the FRET ratio.

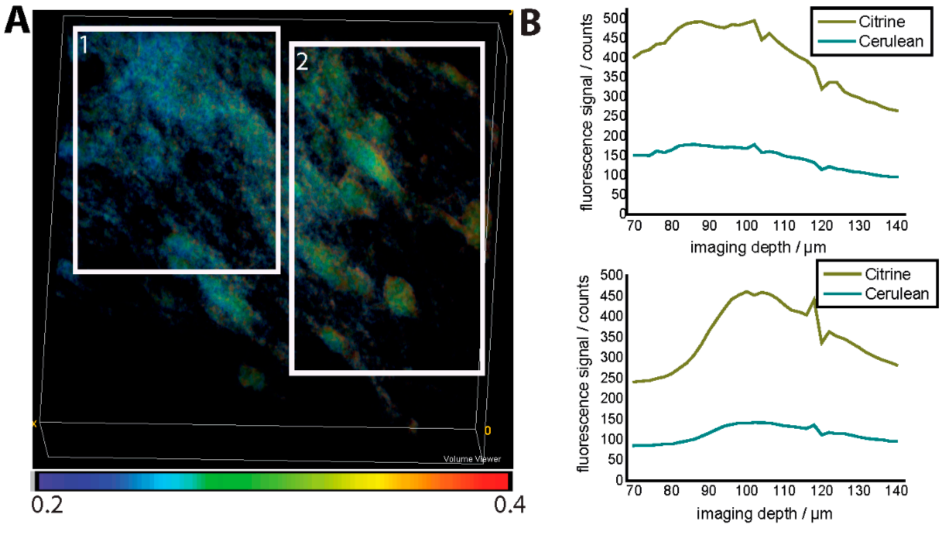

Figure S1. Fluorescence signals of Cerulean and Citrine, respectively, in the brain stem of a healthy, untreated CerTN L15 mouse corresponding to the two different regions in Figure 2A,B.

Movie S1. Time lapse of the NFRET ratio in a 300 × 300 × 70 μm³ region in the brain stem of a *CerTN L15* mouse before and during treatment with KCl. λ_{exc} = 850 nm, λ_{em} (Cerulean) = 475 ± 20 nm, λ_{em} (Citrine) = 535 ± 25 nm.

Movie S2. Time lapse of the Cerulean fluorescence signal in a 300 × 300 × 50 μm³ region in the brain stem of a *CerTN L15* mouse after treatment with KCl. λ_{exc} = 850 nm, λ_{em} (Cerulean) = 475 ± 20 nm.

Movie S3. Time lapse of the Citrine fluorescence signal in a 300 × 300 × 50 μm³ region in the brain stem of a *CerTN L15* mouse after treatment with KCl. λ_{exc} = 850 nm, λ_{em} (Citrine) = 535 ± 25 nm.

Movie S4. Time lapse of the NFRET ratio corresponding to the Cerulean and Citrine signals in Movies S2 and S3.
